# Dentine-derived barrier membrane versus extended platelet-rich fibrin for guided bone regeneration: a 2-year randomized clinical trial

**DOI:** 10.1186/s12903-026-08582-2

**Published:** 2026-05-21

**Authors:** Sally Elsayed Abdelsameaa, Abdullah Maashi Alruwaili, Nesma El-Gohary, Heba Abo-Elfetouh Elsheikh

**Affiliations:** 1 Oral and Maxillofacial Surgery Department, Faculty of Dentistry, Mansoura University, Mansoura, Egypt; 2 Fixed Prosthodontics Department, Faculty of Dentistry, Mansoura University, Mansoura, Egypt

**Keywords:** Immediate dental implant, Dentine-derived barrier membrane, Extended platelet-rich fibrin membrane, Guided bone regeneration

## Abstract

**Background:**

An autogenous dentine-derived barrier membrane (DDBM), primarily consisting of type I collagen, is a recognized as an osteo-conductive material in implant dentistry. A recently developed method is used to prolong the functional properties of PRF to 4 to 6 months. This newly prepared material is called Albumin-platelet rich fibrin (Alb-PRF) or extended platelet-rich fibrin (e-PRF). The aim of this study was to compare clinical and radiographic results of autogenous demineralized dentine-derived barrier membrane versus extended platelet-rich fibrin membrane for guided bone regeneration (GBR) with immediate implant placement in the posterior mandible.

**Materials and methods:**

Forty patients with non-restorable mandibular molars indicated for immediate implant placement and GBR were selected and randomly divided into two equal groups (*n* = 20 per group). In Group 1, implants were placed using a dentine-derived barrier membrane, while in Group 2, implants were placed using an extended platelet-rich fibrin membrane. All patients underwent clinical and radiographic assessments immediately after placement and at 3, 6, 12, and 24 months to evaluate implant stability, peri-implant probing depth (PPD), marginal bone loss (MBL), and relative bone density.

**Results:**

All implants achieved a 100% survival rate over the 24-month period. Implant stability increased significantly over time in both groups (*P* < 0.001), with no significant intergroup differences at any point; at 12 months, the median (IQR) was 78.0 (77.0–79) for Dentine Group and 79 (75.0–81.0) for e-PRF Group (*P* = 0.201, *r* = 0.207). PPD also increased significantly within both groups (*P* < 0.001), but remained within healthy limits, with no significant differences between groups at any points; at 24 months, the median (IQR) was 3.25 mm (3.13–3.50 mm) for Dentine Group vs. 3.25 mm (3.0–3.31 mm) for e-PRF Group; (*P* = 0.142, *r* = 0.241). MBL increased significantly over time in both groups (*P* < 0.001). At 24 months, no significant intergroup differences were found for buccal MBL, the median (IQR) was 1.22 mm (1.20–1.30 mm) for Dentine Group vs. 1.30 mm (1.25–1.39 mm) for e-PRF Group; *P* = 0.060, *r* = 0.300) or lingual MBL, the median (IQR) was 1.27 mm (1.20–1.37 mm) for Dentine Group vs. 1.27 mm (1.19–1.37 mm) for e-PRF Group; (*P* = 0.678, *r* = 0.069). Similarly, relative bone density showed a significant intragroup increase (*P* < 0.001) with no significant intergroup differences at 24 months for buccal density the median (IQR) was 1204.5 (1150.0–1261.0) for Dentine Group vs. 1266.0 (1119.0–1311.0) for e-PRF Group; (*P* = 0.183, *r* = 0.214) or lingual density the median (IQR) was 1180.0 (1156.0–1210) for Dentine Group vs. 1212.50 (1112.0–1233.0) for e-PRF Group; (*P* = 0.314, *r* = 0.163).

**Conclusion:**

Both autogenous DDBM and e-PRF membrane demonstrated favorable clinical and radiographic outcomes over 24 months when used for GBR in conjunction with immediate implant placement in the posterior mandible. Both materials represent cost-effective, biological alternatives for GBR, maintaining stable peri-implant tissues over a 2-year period.

**Trial registration:**

The study was retrospectively registered at ClinicalTrials.gov PRS (https://register.clinicaltrials.gov) under the identifier number NCT07396350 on 02/02/2026.

## Introduction

The alveolar ridge experiences vertical and horizontal resorption after 6 months of tooth extraction, resulting in bone volume reduction and complicating implant placement [1]. This is a primary issue related to tooth extraction. Immediate implant placement is regarded as a reliable and suitable procedure that involves inserting the implant into the newly created extraction socket immediately following tooth extraction. Immediate implant placement is frequently preferred as it reduces surgical and treatment duration and minimizes surgical trauma [2]. A significant clinical challenge of immediate implant placement is the presence of a jumping distance or residual bony defects between the implant surface and the socket walls, which may lead to significant bone resorption and compromised long-term stability. Consequently, the integration of immediate implant insertion with guided bone regeneration (GBR) offers the benefit of reducing marginal bone loss and volumetric resorption during the physiologic remodeling phase of the extraction socket repair [3]. 

GBR’s main concept is the use of membranes to exclude epithelial cells and permit the migration of the desired cells especially osteoblasts derived from the periosteum and/or adjacent bone and/or bone marrow to the bone defect sites [4]. 

Since the identification of the osteogenic potential of the periosteum, membranes have been developed to incorporate the periosteum into bone defects [5]. The following requirements should be met by a barrier membrane: biocompatibility, tissue adhesion without mobility, prevention of soft tissue ingrowth, ease of use, and maintenance of a space [4]. Absorbable and non-absorbable membranes were developed to be used in GBR. The drawbacks of non-absorbable membranes encompass the requirement for additional surgery for removal and their vulnerability to infection upon exposure; nonetheless, they possess a robust mechanical property that promotes osseous proliferation and effective space maintenance [5]. Notwithstanding their inferior mechanical properties, absorbable membranes are extensively utilized for GBR owing to their excellent biocompatibility [5]. 

Bone comprises 70% hydroxyapatite, 18% collagen, 2% non-collagenous proteins, and 10% physiological fluids, similar to dentine [6]. Demineralized dentine matrix (DDM) derived from human teeth is a dense, avascular, and acellular collagen matrix that encompasses non-collagenous growth factors, including bone morphogenic proteins (BMPs), and exhibits significant osteo-inductivity and osteo-conductivity [7]. In implant dentistry, DDM is frequently employed as a bone substitute [8]. Upon introduction into the oral cavity, the mechanical stability of block-type DDM enables it to resist bacterial infiltration and postpone the remodeling process. DDM can be utilized as an osteo-inductive collagen membrane to restore the periosteum [9]. The block-type DDM is used to create dentine derived-barrier membranes (DDBM), which are resorbable, osteo-inductive, and collagenous. DDBM exhibits porosity at both the macro (0.2 mm) and micro levels (dentinal tubules) [9]. 

Platelets or platelet concentrates have been utilized to create several products that function as biological mediators to facilitate healing. Platelet-rich fibrin (PRF) is the second generation that has demonstrated efficacy in therapeutic contexts. It was developed in France by Choukroun and his colleagues [10]. This treatment does not necessitate anticoagulants, bovine thrombin, or other components such as platelet-rich plasma (PRP). PRF’s faster-than-ideal resorption rate, which is within a 2–3 week timeframe, is one of its primary documented disadvantages, so PRF membranes can’t be used as a barrier membrane like collagen because of their rapid resorption rate, which makes it impossible to exclude soft tissues for a long time [11]. In 2015, Kawase et al. [12] presented a heat-compression technique utilizing PRF membranes to enhance its functional characteristics and delay its resorption rate. The heat-compression technique was found to prolong the degradation rate of PRF to beyond 3 weeks.

This technique uses the platelet-poor plasma (PPP) layer, which contains around 60% albumin, and heats it to 75 °C for 10 min in order to denaturize albumin and destroy many weak bonds (such as hydrogen bonds) within its protein molecule. After denaturation, the proteins reorganize into a denser assembly that prolongs PRF’s resorption rate for up to 4 to 6 months. But the heating procedure also kills cells and growth factors, which means that the platelet concentrates lose a lot of their capacity to regenerate. For these reasons, after heating, a concentrated PRF layer (C-PRF) which preserves viable platelets and leukocytes extracted from the buffy coat is mixed back into the heated PPP (albumin gel) after cooling to yield albumin-PRF (Alb-PRF) or extended PRF (e-PRF). The resulting fibrin–albumin scaffold provides a sustained release of key growth factors [11]. 

Current trends in regenerative dentistry show a growing clinical interest in utilizing entirely biological and autogenous materials for GBR. DDBM and e-PRF membrane represent two distinct biological membranes. Their comparative performance in maintaining bone volume at immediate implant sites remains largely unexplored. Therefore, this study aimed to compare the clinical and radiographic outcomes of autogenous DDBM versus e-PRF membrane for GBR with immediate implant placement in the posterior mandible. The null hypothesis of this trial was that there would be no statistically significant differences at the 24-month follow-up between autogenous DDBM and e-PRF membrane used in immediate posterior mandibular implants regarding the primary outcome of marginal bone loss (MBL) and relative bone density, or the secondary outcomes of implant stability and peri-implant pocket depth.

## Materials and methods

The study included 40 patients recruited from the outpatient clinic of the oral and maxillofacial surgery department, Faculty of Dentistry, Mansoura University. These patients had non-restorable mandibular molars that were extracted and replaced with immediate dental implants. This prospective randomized clinical trial was conducted following ethical approval from the Ethical Committee of the Faculty of Dentistry, Mansoura University (Approval No. R.26.01.96) in accordance with the seventh revision of the Declaration of Helsinki (2013).

All patients provided written informed consent after being made aware of the procedure’s possible risks and benefits.

### Selection of patients

The subsequent criteria were employed to choose the patients for the study:

Eligibility criteria:

1. Patients aged 18 years and older.
2. Non-restorable mandibular molars requiring extraction and GBR for immediate implant placement.
3. The tooth that required extraction showed no clinical or radiographic signs of acute infection.
4. There was at least 5 mm of available bone between the superior border of the inferior alveolar canal and the root apex.
5. At least 8 mm inter-arch space for the prosthesis.
6. Patients were free of systemic conditions that absolutely contraindicate implant insertion.
7. Patients with good oral hygiene.
8. Patients capable of adhering to the mandated follow-up appointments.

Exclusion criteria:

1. Tobacco smokers.
2. Pregnancy.
3. Patients with a history of radiation to the head and neck.
4. Bruxism and parafunctional habits.

### Calculation of sample size

The mean bone density data from a previous study by Mizar et al. [13] served as the basis for calculating the sample size. Utilizing the G Power program version 3.1.9.7, the sample size was calculated based on an effect size of 1.06, employing a two-tailed test, with an α error of 0.05 and a power of 90.0%. Consequently, the total calculated sample size was 20 implants per group, resulting in a total of 40 implants for the study.

### Randomization and allocation

A simple lottery randomization approach was used to randomly allocate the study participants into two equal groups (1:1 allocation ratio), with 20 implants assigned to each group. The resulting assignments were secured in sequentially numbered, opaque, sealed envelopes. To ensure allocation concealment, these envelopes were managed by an independent staff member not involved in the surgical procedures. The treatment group was revealed to the surgeon immediately before the intervention.

### Blinding

Blinding of the operator and patients were unfeasible due to the nature of the intervention. To mitigate potential bias, outcome evaluation was conducted by an independent assessor who was not involved in the surgical phase and remained unaware of the group allocation. Additionally, the statistician was blinded to group assignments; data were provided using generic codes (Group 1 and Group 2), which were only unmasked after the completion of the statistical analysis.

### Study design

**Fig. 1:**
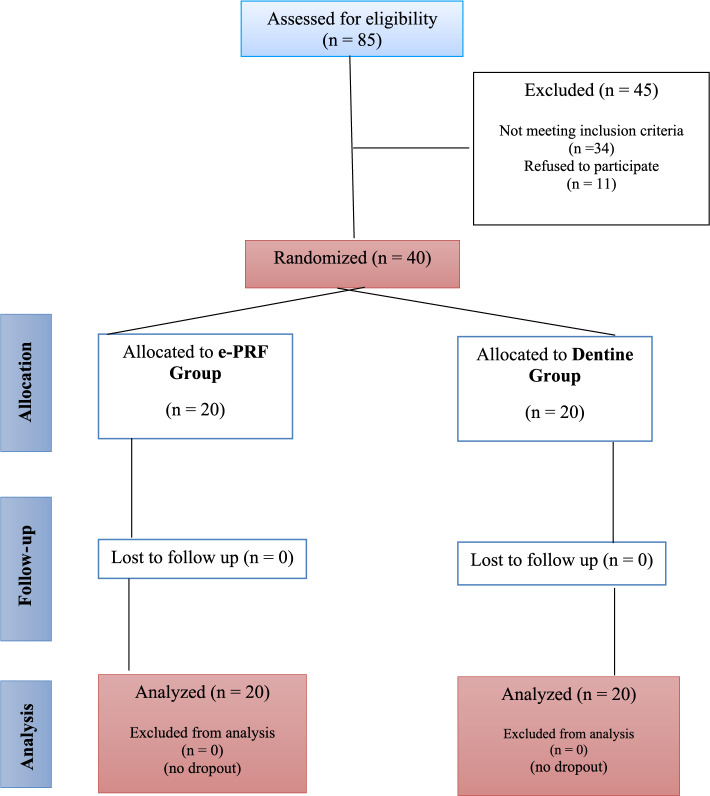
CONSORT Flowchart

This prospective randomized clinical study was executed in compliance with the CONSORT guidelines for clinical trial CONSORT Flowchart (Fig. 1).

Two equivalent groups were randomly assigned to this trial:

- Group 1: 20 immediate implants were inserted using autogenous DDBM.
- Group 2: 20 immediate implants were inserted using e-PRF membrane.

### Preoperative evaluation and assessments

#### Clinical evaluation

Comprehensive medical and dental histories were obtained, succeeded by extraoral and intraoral examinations. The tooth indicated for replacement was assessed to confirm the lack of acute infection. Study casts were mounted on semi-adjustable articulators to assess occlusion, crown height space, and tooth inclination.

**Fig. 2:**
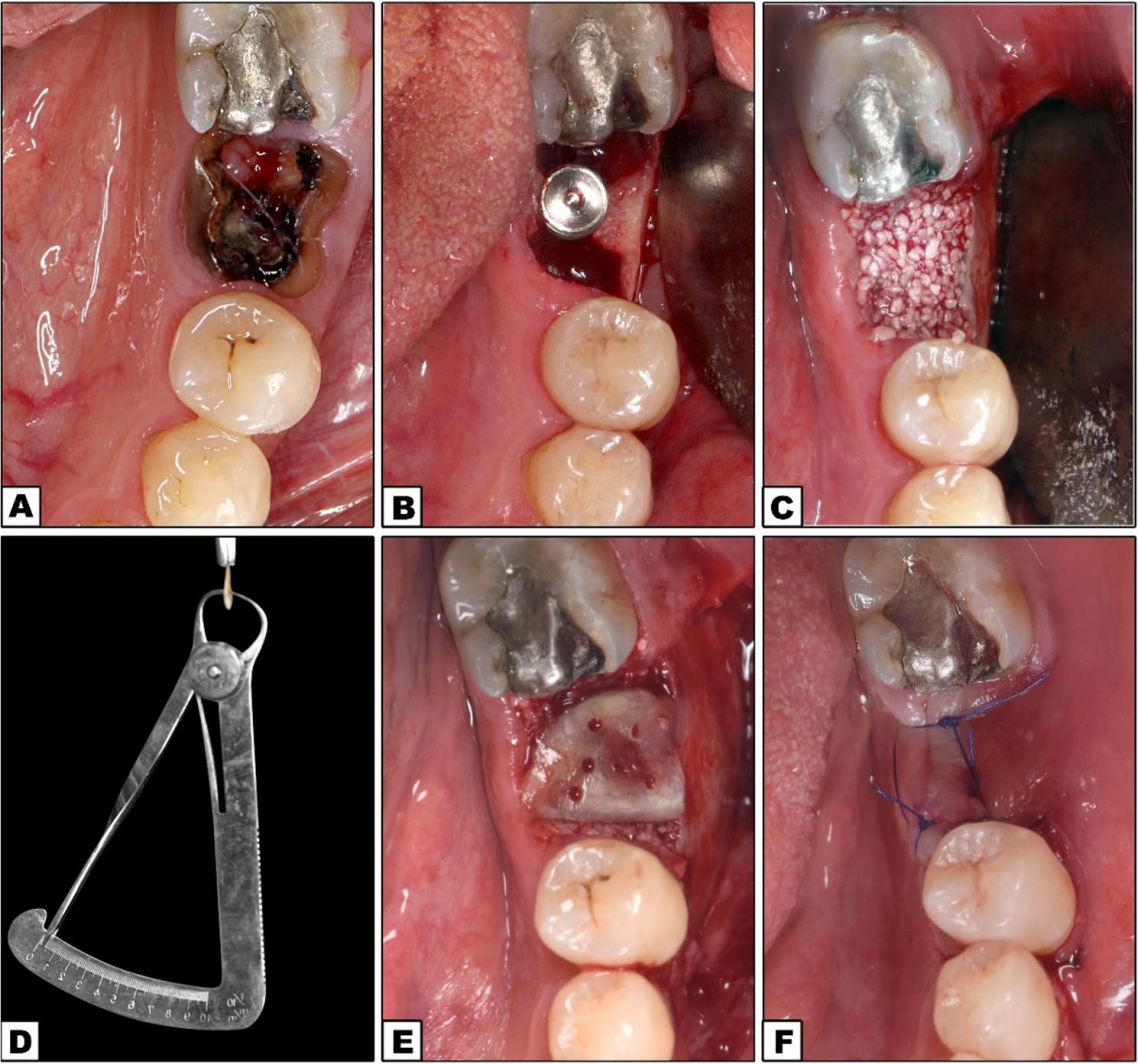
DDBM Group. **A** Preoperative occlusal view of non-restorable lower left 1st molar; **B** Implant insertion; **C** Allograft application; **D** DDBM preparation; **E** DDBM application; **F** Flap closure and suturing

**Fig. 3:**
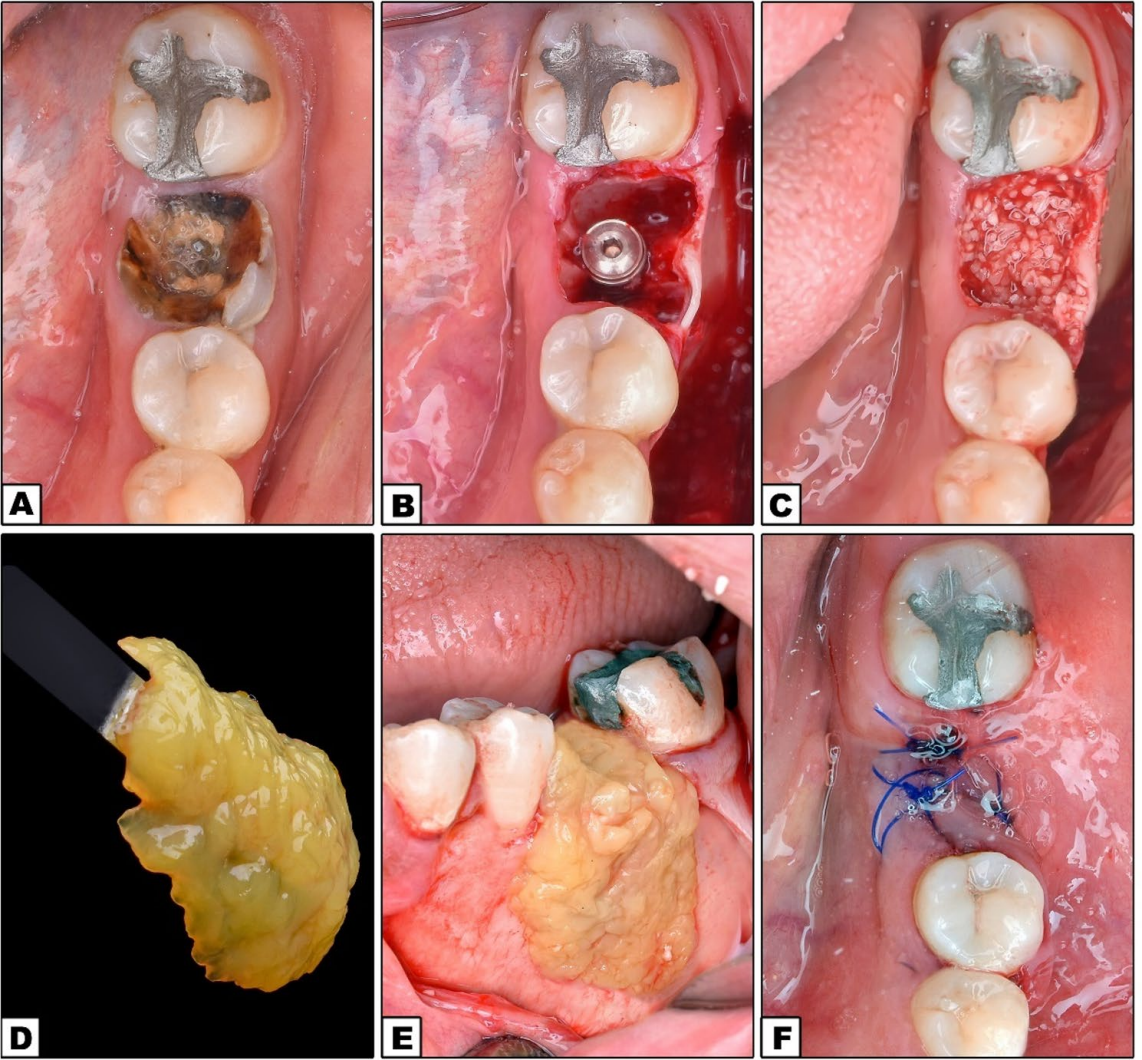
e-PRF Group. **A** Preoperative occlusal view of non-restorable lower left 1st molar; **B** Implant insertion; **C** allograft application; **D** e-PRF membrane; **E** e-PRF application; **F** Flap closure and suturing

Preoperative photos were captured as a baseline record (Figs. 2A and 3A). CBCT was conducted for virtual implant planning, encompassing the implant’s position, length, and diameter (Figs. 5A and 6A).

Two days prior to surgery, Amoxicillin with clavulanic acid 1gm (Augmentin, GlaxoSmithKline, U.K.) was administered every 12 h as a prophylactic antibiotic, and the patient was instructed to rinse with chlorhexidine antiseptic mouthwash (Orovex, Macro Pharmaceuticals, Egypt) for 30 s before the procedure.

#### Operative phase

The surgery was performed using local anesthesia (2% mepivacaine with 1: 20,000 levonordefrin) (2% mepivacaine HCL with 1:20,000 levonordefrin, Alexandria Co. for Pharmaceuticals and Chemical Industries, Alexandria, Egypt). Following the administration of local anesthesia, a three-line incision was made, then the mucoperiosteal flap was reflected. Mandibular molars were sectioned using a high-speed surgical bur, and appropriate elevators were employed to atrumatically extract the roots individually, ensuring the preservation of the buccal and lingual bone plates. The socket was then curetted and properly irrigated to remove any granulation tissue. Periosteal release incisions were made at the deepest point of the mucoperiosteal flap in the buccal region of the mandible to ensure tension free closure of the flap. The initial pointed drill was employed to create a hole. Then, the drills of progressively larger diameters were used to finalize the osteotomy in accordance with the manufacturer’s instructions. Implants (IS II Active, Neobiotech^®^, Seoul, South Korea) were inserted into the prepared osteotomies, engaging the bone apical to the socket to obtain sufficient primary stability with a minimum insertion torque of 35 Ncm (Figs. 2B and 3B). According to preoperative CBCT, implant lengths ranged from 10 to 11.5 mm and diameters ranged from 4 to 5 mm.

The implant platform was positioned 5 mm apically from the free gingival margin. Allograft bone grafting material (Demineralized Cortical Cancellous Powder < 2 mm) (OneAllo Graft, OneGraft, Austria) was used to fill the gap around the implant and cover any peri-implant bone defect (Figs. 2C and 3C).

### For Group 1

#### Autogenous DDBM preparation[9]

The extracted tooth was immersed in 75% alcohol, and any residual periodontal tissue was removed, then layers of enamel and cementum were removed with a rotary instrument (Diamond bur). The pulp tissue and root canal filling were both removed. A micro-fissure bur was used to create holes from the dentine surface to the pulp chamber, each measuring 0.2 mm in diameter. The tooth was then dissected in the occlusal-apical direction to create a membrane with a thickness of 300 to 800 μm. To partially demineralize the dentine, DDBM was submerged in a 10% EDTA solution for three minutes. As a result, active growth factors were released and the collagen fibers were exposed. The membrane was then cleaned once again using a buffered saline solution. Without using any fixation technique, DDBM was carefully positioned to cover the alveolar socket up to the height of the lingual and buccal plates (Fig. 2D and E).

### For Group 2

#### Preparation of an e-PRF[11]

**Fig. 4:**
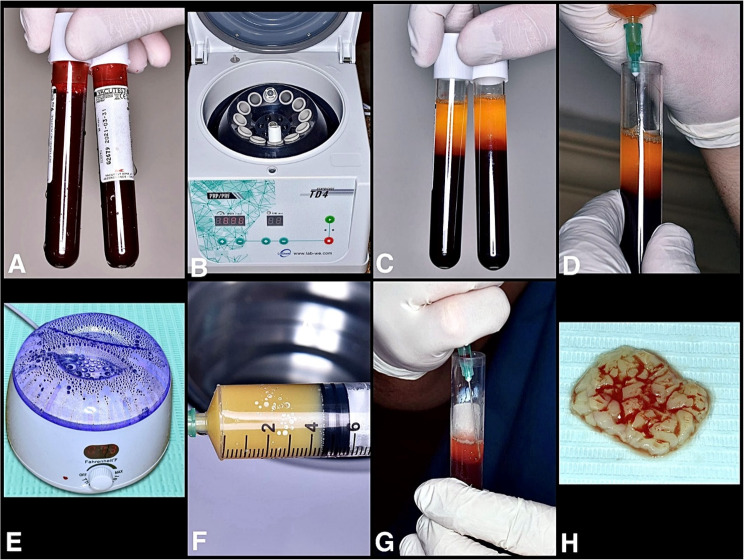
e-PRF membrane preparation. **A** Peripheral blood; **B** Centrifuge Device; **C** Blood plasma and remaining decanted red cells; **D** Platelet-poor plasma collection with a syringe to be placed into the heat device; **E** Heating device; **F** Albumin gel (**G**) Liquid PRF collection; **H** e-PRF membrane

Peripheral blood was collected in 9–10 mL tubes without any additives (Fig. 4A). The blood tubes were positioned in a centrifuge at 700 g RCF for a duration of 8 min (TD4 PRP/ PRF Portable Low-Speed Centrifuge, China) (Fig. 4B). After processing, the blood was separated into plasma and decanted red cells (Fig. 4C). Two to four milliliters of plasma (platelet-poor plasma) were extracted using a syringe and heated for 10 min at 75 °C, while the residual blood components (buffy coat, liquid PRF, and red blood cells) were placed in a cooling device (Fig. 4D and E). Following 10 min of heating, the PPP layer transformed into Albumin gel (Fig. 4F) The Albumin gel was subsequently cooled in the cooling device for 1–2 min. Once cooled, the gel was transferred into a custom-sized tray. The remaining 1–2 mL of concentrated liquid-PRF layer was collected and placed into the same custom-sized tray with the Albumin gel, allowing them to set together for 15 min to form e-PRF membrane (Fig. 4G and H) The e-PRF membrane was then used to cover the augmented area (Fig. 3D and E).

### For both groups

The periosteal flap was repositioned, and primary closure was achieved utilizing horizontal mattress and interrupted 4/0 polypropylene sutures (Figs. 2F and 3F).

### Postoperative care and instructions

Patients were instructed to apply cold compresses for the first 24 h. Starting the day after surgery, they were advised to use a chlorhexidine mouthwash to maintain proper oral hygiene. Postoperatively, patients continued a regimen of amoxicillin/clavulanic acid (1 g every 12 h) for five days to prevent infection. Additionally, diclofenac potassium (50 mg; Cataflam, Alexandria Pharmaceutical and Chemical Industries, Egypt) was prescribed every 8 h for five days for pain management. Sutures were removed after 14 days.

### Prosthetic phase

The implants were loaded after 3 months with final restorations.

#### Evaluation

All measurements were performed by a single experienced examiner blinded to the group allocation.

A- Clinical evaluation:

- 1 - Implant Stability.

The implant stability was evaluated immediately postoperatively and after 3, 6, and 12 months. An Osstell Mentor device (Osstell, Savadaled, Sweden; Integration Diagnostics) was utilized to assess implant stability utilizing resonance frequency analysis (RFA). Measurements were made at 90° in four different directions in order to calculate the RFA value. The implant stability quotient (ISQ) was calculated by averaging the results for each implant [14].

- 2 – Peri-implant Pocket Depth (PPD).

The distance between the base of the implant sulcus and the gingival margin was measured using a calibrated plastic implant probe [15]. 

**Fig. 5:**
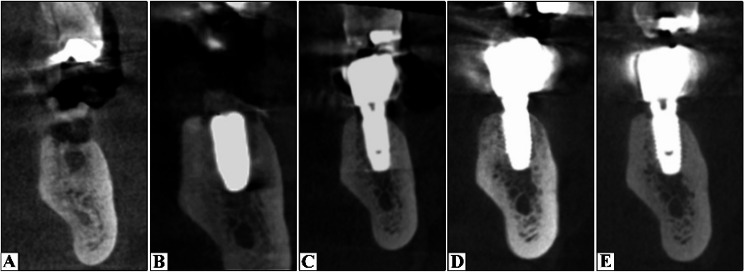
Radiographic Evaluation of DDBM group. **A** Preoperative; **B** Immediate postoperative; **C** 3 months postoperative (At loading); **D** 12 months postoperative; **F** 24 months postoperative

**Fig. 6:**
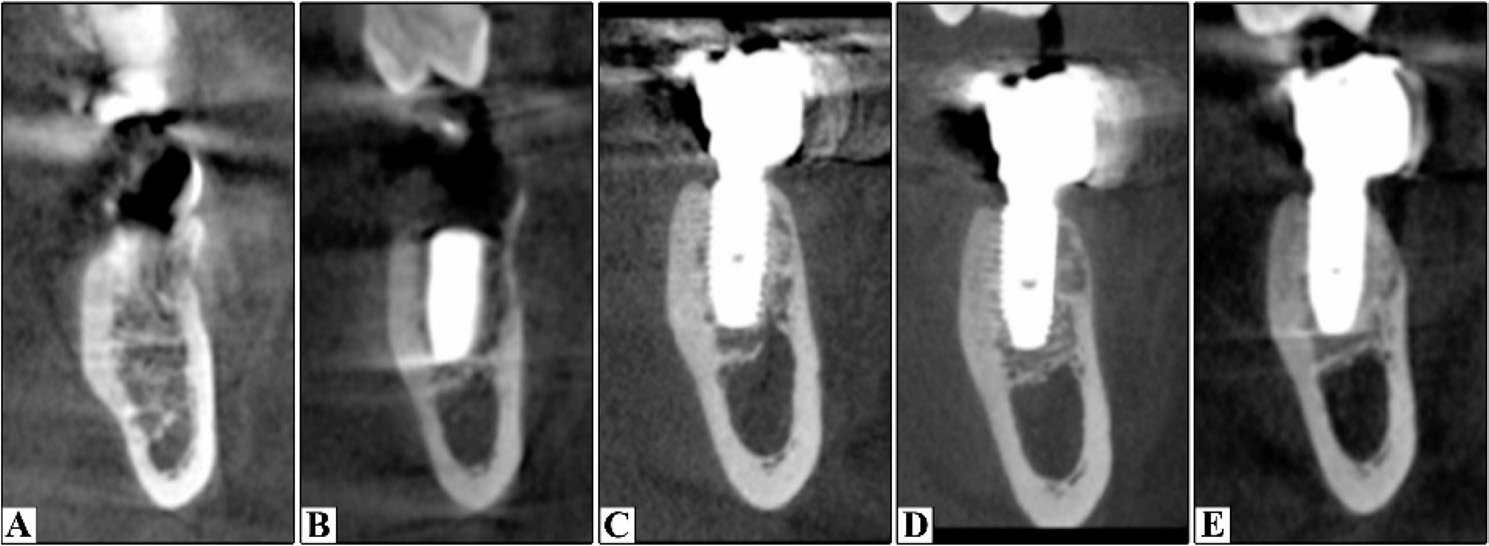
Radiographic Evaluation of e-PRF group. **A** Preoperative; **B** Immediate postoperative; **C** 3 months postoperative (At loading); **D** 12 months postoperative; **F** 24 months postoperative

B- Radiological Evaluation:

Cone Beam Computed Tomography (CBCT) examinations were obtained at a single radiology center using the same imaging system (Planmeca ProMax^®^ 3D Max, Helsinki, Finland) and standardized exposure settings (89 kVp, 8 mA). Scans were acquired immediately after surgery and at 3-, 12-, and 24-month follow-up intervals. All images were processed and reconstructed using OnDemand3D software (Figs. 5 and 6). All measurements were performed three times, and the mean value was calculated to minimize measurement error.

- 1- Marginal Bone Loss (MBL).

The implant’s cross-section was adjusted at the center to establish a reference for assessing MBL. In the cross-sectional image, a line was drawn parallel to the implant, starting at the crest of the buccal bone plate and terminating at the implant’s apex [16]. Height was measured immediately postoperatively and after 3, 12, and 24 months. The same procedure was employed in the lingual side. The bone height that was measured immediately postoperatively was considered the baseline value. Radiographic MBL was determined as the difference between measurements taken at 3, 12 and 24 months and the baseline value.

- 2-Relative Bone Density.

The greyscale bone measuring tool was used to calculate relative bone density from the bucco-lingual aspect of the cross-sectional view. Six measurements were obtained for each implant, and the mean value was calculated. The assessments were conducted 1 mm parallel to the implant fixture. Three readings were recorded on the buccal aspect of the implant fixture (coronal, middle, and apical thirds), followed by three readings on the lingual side (in the same sequence) [17]. Relative bone density was measured at baseline and at 3, 12, 24 months.

### Statistical analysis

Data were analyzed using the IBM SPSS software package, version 27.0 (IBM Corp., Armonk, NY, USA; released 2020). Categorical data were summarized as frequencies and percentages, and the Chi-square test was applied for intergroup comparisons. For continuous data, the normality of distribution was assessed using the Shapiro-Wilk test, skewness, Q-Q plots, and histograms. Since the quantitative variables were non-normally distributed, they were expressed as median and interquartile range (IQR). The Mann-Whitney U test was used to compare differences between the two independent groups at each time point, while the Friedman test was utilized for longitudinal comparisons within each group across the study periods. To determine the magnitude of the difference between groups, the effect size (r) was calculated alongside 95% confidence intervals (CI) and interpreted as small (< 0.30), moderate (0.30–<0.50), or large (≥ 0.50). All tests were two-tailed, and statistical significance was set at the 5% level (*P* ≤ 0.05).

### Result

Forty patients, aged from 21 to 58 years, were included in this study and each patient received only one implant. In the Dentine membrane Group, there were 8 males (40%) and 12 females (60%), whereas in the e-PRF membrane Group, there were 6 males (30%) and 14 females (70%). 4 s molars (10%) and 36 first molars (90%) were restored with immediate dental implants, with a total of 40 implants. Implant length ranged from 10 to 11.5 mm and diameter ranged from 4 to 5 mm. Over the course of the follow-up period, all implants had a 100% success rate with no documented complications (Table 1).

**Table 1:** Demographic data and clinical characteristics of dental implant

Parameter			e-PRF (*n* = 20)		Dentine (*n* = 20)		*P*-value
Age (years)	Mean ± SD		35.90 ± 10.62		34.60 ± 5.42		0.619
	Range		21–58		26–42		
Sex	Female		14(70.0)		12(60.0)		0.507
	Male		6(30.0)		8(40.0)		
Site	First molar		18 (90.0)		18(90.0)		1.000
	Second molar		2(10.0)		2(10.0)		
Implant Diameter	Mean ± SD		4.60 ± 0.32		4.55 ± 0.28		0.592
	Range		4.0–5.0		4.0–5.0		
Implant length	Mean ± SD	10.30 ± 0.62		10.38 ± 0.67		0.697	
	Range	10–11.5		10–11.5			

Between-group comparisons for continuous variables (Age, Implant Diameter, Implant Length) used independent t-tests. Categorical variables (Sex, Site) used chi-square tests

*SD* standard deviation

*P* ≤ 0.05 is statistically significant

### Complications and wound healing

No intraoperative or postoperative complications were recorded for any of the 40 patients. All surgical wounds achieved healing by primary intention without evidence of flap dehiscence or premature membrane exposure. Furthermore, no signs of infection, suppuration, or persistent swelling were observed during the follow-up period in either group.

A- Clinical evaluation:

- 1- Implant Stability.

Implant stability showed no statistically significant differences between the two groups at implant insertion or at 3, 6, and 12 months (*P* = 0.968, 0.165, 0.369, and 0.201, respectively), with effect size estimates indicating small between-group differences across all time points. However, within each group, implant stability increased significantly over time (*P* < 0.001) reflecting a marked and clinically relevant improvement in implant stability throughout the follow-up period (Table 2).

**Table 2:** Implant stability

Implant stability	e-PRF (*n* = 20)	Dentine (*n* = 20)	*P*	Effect size *r* (95% CI)
At Surgical time	69.50 (67.0–72.0)	70.0 (68.0–71.0)	0.968	0.009 (-0.304–0.319)
3 months	70.0 (69.0–73.0)	72.0 (71.0–73.0)	0.165	0.224 (-0.094–0.500)
6 months	73.50 (72.0–76.0)	73.0 (73.0–74.0)	0.369	0.149 (-0.170–0.440)
12 months	79.0 (75.0–81.0)	78.0 (77.0–79.0)	0.201	0.207 (-0.111–0.487)
P_0_	P_0_<0.001^*^	P_0_<0.001^*^		

Data was expressed using Median (IQR)

*IQR* Inter quartile range, *CI* Confidence interval

P: *P* value for Mann Whitney test for comparing between e-PRF and Dentine

P_0_: *P* value for Friedman test for comparing between different time points in each group

*: Statistically significant at *P* ≤ 0.05 (All tests were two-tailed)

- 2 – Peri-implant Pocket Depth (PPD).

PPD showed no statistically significant differences between the two groups at any evaluated time point (*P* > 0.05), with effect size estimates indicating small between-group differences. In contrast, within each group, median PPD values increased significantly over the study period (*P* < 0.001) (Table 3).

**Table 3:** Peri-implant Pocket Depth (PPD)

Peri-implant pocket depth	e-PRF (*n* = 20)	Dentine (*n* = 20)	*P*	Effect size *r* (95% CI)
3 months	1.63 (1.63–1.63)	1.69 (1.50–1.75)	0.698	0.067 (-0.250–0.371)
12 months	2.38 (2.25–2.50)	2.31 (2.13–2.50)	0.602	0.089 (-0.229–0.389)
24 months	3.25 (3.0–3.31)	3.25 (3.13–3.50)	0.142	0.241 (-0.076–0.514)
**P**_**0**_	P_0_<0.001^*^	P_0_<0.001^*^		

Data was expressed using Median (IQR)

*IQR* Inter quartile range, *CI* Confidence interval

P: *P* value for Mann Whitney test for comparing between e-PRF and Dentine

P_0_: *P* value for Friedman test for comparing between different time points in each group

*: Statistically significant at *P* ≤ 0.05 (All tests were two-tailed)

B-Radiological Assessment.

- 1- Marginal Bone Loss (MBL).

For buccal MBL, no statistically significant differences were observed between the two groups at any evaluated time point (*P* > 0.05), with effect size estimates indicating small between-group differences. For lingual MBL, no significant differences were found at 3 and 24 months (*P* = 0.429 and 0.678, respectively), with small effect sizes; however, a statistically significant difference was detected at 12 months (*P* = 0.002), with a corresponding moderate effect size. Within each group, both buccal and lingual median MBL values increased significantly over the follow-up period (*P* < 0.001). (Table 4)

**Table 4:** Marginal bone loss (MBL)

Marginal bone loss (MBL)	e-PRF (*n* = 20)	Dentine (*n* = 20)	*P*	Effect size *r* (95% CI)
Buccal				
3 months	0.43 (0.38–0.53)	0.42 (0.35–0.47)	0.429	0.129 (-0.191–0.423)
12 months	1.03 (0.98–1.07)	1.01 (0.90–1.05)	0.201	0.206 (-0.112–0.487)
24 months	1.30 (1.25–1.39)	1.22 (1.20–1.30)	0.060	0.300 (-0.013–0.559)
P_0_	P_0_<0.001^*^	P_0_<0.001^*^		
Lingual				
3 months	0.39 (0.35–0.43)	0.41 (0.37–0.51)	0.429	0.129 (-0.190–0.423)
12 months	1.01 (0.97–1.04)	1.06 (1.03–1.13)	0.002^*^	0.490 (0.210–0.695)
24 months	1.27 (1.19–1.37)	1.27 (1.20–1.37)	0.678	0.069 (-0.248–0.372)
P_0_	P_0_<0.001^*^	P_0_<0.001^*^		

Data was expressed using Median (IQR)

*IQR* Inter quartile range *CI* Confidence interval

P: *P* value for Mann Whitney test for comparing between e-PRF and Dentine

P_0_: *P* value for Friedman test for comparing between different time points in each group

*: Statistically significant at *P* ≤ 0.05 (All tests were two-tailed)

- 2-Relative Bone Density.

For buccal relative bone density, no statistically significant differences were observed between the two groups at any evaluated time point (*P* > 0.05), with effect size estimates indicating small between-group differences. For lingual relative bone density, no significant differences were found at the time of implant insertion and at 12 and 24 months (*P* = 0.121, 0.201, and 0.314, respectively), with small effect sizes; however, a statistically significant difference was detected at 3 months (*P* = 0.040), with a corresponding moderate effect size. Within each group, both buccal and lingual relative bone density values increased significantly over the follow-up period (*P* < 0.001) (Table 5).

**Table 5:** Relative bone density

Bone density	e-PRF (*n* = 20)	Dentine (*n* = 20)	*P*	Effect size *r* (95% CI)
Buccal				
After placement	886.5(738.0–917.0)	861.0(790.0–950.0)	0.841	0.034 (-0.280–0.342)
3 months	1123.0(949.0–1265.0)	1040.5(989.0–1100.0)	0.242	0.188 (-0.131–0.472)
12 months	1213.0(1105.0–1267.0)	1173.0(1075.0–1193.0)	0.052	0.308 (-0.003–0.566)
24 months	1266.0(1119.0–1311.0)	1204.5(1150.0–1261.0)	0.183	0.214 (-0.104–0.493)
P_0_	P_0_<0.001^*^	P_0_<0.001^*^		
Lingual				
After placement	826.5(810.0–866.0)	770.0(699.0–823.0)	0.121	0.248 (-0.068–0.520)
3 months	1063.5(1016.0–1152.0)	964.0(900.0–1017.0)	0.040^*^	0.325 (0.015–0.578)
12 months	1163.5(1046.0–1207.0)	1120.0(1100.0–1158.0)	0.201	0.205 (-0.113–0.486)
24 months	1212.5(1112.0–1233.0)	1180.0(1156.0–1210.0)	0.314	0.163 (-0.157–0.451)
P_0_	P_0_<0.001^*^	P_0_<0.001^*^		

Data was expressed using Median (IQR)

*IQR* Inter quartile range, *CI* Confidence interval

P: *P* value for Mann Whitney test for comparing between e-PRF and Dentine

P0: *P* value for Friedman test for comparing between different time points in each group

*: Statistically significant at *P* ≤ 0.05 (All tests were two-tailed)

## Discussion

Immediate implant insertion has emerged as a favored therapeutic option for individuals in rehabilitation. This approach offers numerous benefits, including reduced surgical interventions, diminished discomfort and morbidity, and shorter treatment times [18]. However, immediate implant placement in the lower molar region presents challenges due to anatomical considerations such as the inferior alveolar nerve and limited bone availability for implant stability. Additionally, the multi-rooted nature of molars results in extraction sockets that exceed the diameter of the implant, creating a gap between the implant’s cervical region and the surrounding bone [19]. 

The purpose of this study was to evaluate the clinical and radiographic outcomes of autogenous DDBM against e-PRF membrane for GBR after immediate implant placement in lower molars.

The implants utilized in this study attained a 100% survival rate, attributable to factors like meticulous patient selection, adherence to oral hygiene protocols during the follow-up period, and the implementation of suitable surgical interventions.

Resorbable barrier membranes are the commonly used biomaterial to cover the augmented area in GBR in order to guarantee graft stabilization and to prevent soft tissue ingrowth [20]. Using a combination of a resorbable membrane and bone graft would considerably preserve the total soft tissue and bone dimensions compared to using a membrane alone [21]. 

The clinical utility of tooth-derived materials has traditionally centered on the use of particulate grafts for defect filling and socket preservation [22–24], or shell techniques for ridge augmentation [25, 26]. However, the clinical application of DDBM specifically as a barrier membrane remains under-reported. To date, only a single study has compared DDBM directly against the conventional collagen membrane, suggesting that DDBM may represent a promising, autogenous alternative in GBR procedures [27]. 

The regenerative capacity of dentine is attributed to its high concentration of essential growth factors, specifically transforming growth factor-beta (TGF-β), insulin-like growth factor-II (IGF-II), and bone morphogenetic protein-2 (BMP-2). Its specialized preparation has been shown to maximize cell proliferation, upregulate key osteogenic genes and accelerate the formation of phosphate nodules, confirming the superior osteogenic potential of dentine [28]. BMPs, osteopontine, dentine sialoproteins, bone connexins, and alkaline phosphatase are all present in the DDM. These proteins are essential for both bone development and bone calcification. DDM can promote alveolar bone regeneration in reconstructive procedures because it can attract osteoblasts and osteoprogenitor cells [29]. 

DDBM is derived from teeth that have been immersed in 75% alcohol to ensure thorough disinfection [30]. DDBM revealed a natural Type 1 collagen membrane that is biomimetic of cortical bone because of the mineral component that remains after demineralization. DDBM has macropores that range from 0.2 to 0.3 mm and innate micropores (dentinal tubules) that range from 3 to 5 μm. A greater hole size typically indicates higher cell and nutrient penetration and tissue-occlusivity in membranes that have been found to be crucial for bone regeneration through the diffusion of cells, growth proteins, and blood circulation with nutrients [9]. The membrane has a thickness of 300 to 800 μm. This thickness ensures adequate rigidity for space maintenance, so DDBM has the potential to be an osteo-inductive collagen membrane [9]. 

One of the main disadvantages of PRF is its short resorption period (2 weeks), which makes it incapable of preventing soft tissue invasion over an extended period of time similar to collagen membrane. The biological resorption capabilities can be extended from a typical 2 weeks to 4–6 months by heating the PPP layer from liquid-PRF. As a result, e-PRF can be employed in place of conventional collagen membranes, which are commonly used in implant dentistry and periodontology [11]. The versatility of e-PRF as a substitute for collagen membranes is supported by its successful implementation in various procedures such as socket grafting, complex GBR, sinus augmentation and immediate implant placement [11, 31–33]. e-PRF continuously releases growth factors. Vascular Endothelial Growth Factor (VEGF) encourages angiogenesis, Insulin-like growth factor (IGF) increases the osteogenic response, while Platelet-derived growth factor (PDGF) and Transforming growth factor (TGF) stimulate osteoblast/mesenchymal stem cell proliferation and differentiation, all of which improve bone production [34]. 

In the present study, allografts were utilized in both groups to fill the peri-implant gap. Allografts are recognized for their dual osteoconductive and osteoinductive properties, which are essential for maintaining osseous volume and limiting vertical bone loss and resorption of the buccal plate during the healing phase of immediate implants [35]. 

The current study revealed that both groups exhibited similar implant stability across all postoperative intervals, with no statistically significant differences observed immediately after placement or at 3, 6, and 12 months postoperatively. High primary stability in both groups can be explained due to presence of sufficient bone apical to extraction socket to be engaged by the implant. Also, presence of adequate inter-radicular bone after osteotomy site preparation is essential for achieving high primary stability [36]. After 3 months the secondary stability increased in both groups. This result suggests that using DDBM and e-PRF membrane may accelerate the implant osseointegration process by maintaining a bioactive environment that facilitates de novo bone formation at the implant-tissue interface, thereby positively influencing the biological stability profile during the early healing phase. This finding is in line with Khalifah et al. [37] and Pirpir et al. [38] Similarly, Mohamed et al. [39] had high primary and secondary stability when used concentrated growth factors around immediate dental implant.

Regarding PPD, no statistically significant differences were observed between the two groups (*P* > 0.05). In both groups, PPD values were less than 3.5 mm after 24 months, indicating that DDBM and e-PRF membrane provide healthy peri-implant condition. These values are below the 6-mm value reported by Monje and Salvi [40], who stated that a distance exceeding 6 mm between the gingival margin and the base of implant sulcus is an indication of per-implantitis. The statistically significant increase in PPD observed between 3 and 24 months should be interpreted as a physiological maturation of the peri-implant tissue rather than a pathological event. Several factors contribute to this trend. First, the elevation of a full-thickness mucoperiosteal flap results in a more apically positioned junctional epithelium during the healing phase. Additionally, because the vascular structure of the peri-implant mucosa is compromised, open wounds heal more slowly [41]. Second, the implant platform was positioned 5 mm apical to the free gingival margin to facilitate the development of an ideal emergence profile and ensure sufficient vertical space for the establishment of the biological width (supracrestal tissue attachment). Similarly, Alsabahi et al. [33] had similar results when used Alb-PRF membrane for immediate dental implant placement in the maxillary esthetic zone. Similar results were also reported by Abdel-Rahman et al. [41]., who used PRF membranes with immediate implants. Our study aligns with the findings of Ali and Khalil [42], El Komi et al. [43] and Chauhan et al. [44].

Concerning MBL, both groups demonstrated acceptable MBL from implant placement to 2-year evaluation, with no statistically significant differences between them. This indicates that both DDBM and e-PRF membrane led to a better coronal position of the alveolar bone crest. MBL of 1.5 to 2 mm during the first year of functional loading and an annual 0.2 mm bone loss was deemed acceptable, according to previous study [45]. The MBL values observed in this study are consistent with findings from Wu et al. [46] and Alhaj et al. [47] who similarly reported limited MBL around immediate implants.

Regarding relative bone density both groups revealed a significant increase over time (*P* < 0.001). No statistically significant differences were observed after 24 months (*P* > 0.05) when comparing both groups. This effect could be explained by the fact that DDBM can attract osteoblasts and osteoprogenitor cells, whereas e-PRF releases growth factors continuously, enhancing the osteogenic response and promoting the growth and differentiation of mesenchymal stem cells and osteoblasts, which in turn increases bone formation [29, 34]. Our study is in line with Abulhassan et al. [2], who reported a bone density increase exceeding 15% in the freshly demineralized dentine graft group when used with an immediate implant of mandibular molars. Our findings were consistent with those of Issa et al. [48], who found that the implant’s bone density increased 3 months after insertion and kept on increasing 2 months after the prosthesis was placed.

### Limitations

Even though the 24-month follow-up period yields valuable mid-term data, this prospective randomized clinical trial has some limitations. First, a non-grafted control group is necessary to fully elucidate the magnitude of the regenerative effect of these membranes. Second, the generalizability of the results is limited by the study’s single-center design. While this design ensured strict control over surgical and prosthetic protocols—thereby strengthening internal validity—it may limit the external validity of the findings. Third, although the sample size was determined based on a power calculation, a total of 40 implants is relatively small to detect rare complications or subtle clinical differences. Consequently, multi-center studies with larger and more diverse populations are necessary to confirm their broader clinical relevance. Fourth, while all assessments were performed by a blinded and highly experienced examiner, formal intra-rater and inter-rater reliability for clinical and radiographic measurements was not formally recorded. Lastly, longer follow-up periods (e.g., 5–10 years) are required to establish the long-term stability of peri-implant tissue and the maintained efficacy of both barrier membranes.

## Conclusion

Within the limitations of the present study, both autogenous DDBM and e-PRF membranes showed favorable clinical and radiographic outcomes over the 24-month follow-up period, with no statistically significant differences detected between the two groups. Although their use requires additional chairside preparation time, both membranes can be considered cost-effective biological resorbable membranes.

## Data Availability

The corresponding author can provide the data sets utilized and/or analyzed for this study upon reasonable request.

## Abbreviations

GBR: Guided Bone Regeneration
DDM: Demineralized Dentine Matrix
DDBM: Dentine-Derived Barrier Membrane
PRF: Platelet-rich fibrin
PPP: Platelet-poor plasma
C-PRF: Concentrated platelet-rich fibrin
Alb-PRF: Albumin platelet-rich fibrin
e-PRF: Extended platelet-rich fibrin
CBCT: Cone Beam Computed Tomography
RFA: Resonance Frequency Analysis
ISQ: Implant Stability Quotient
PPD: Peri-implant Pocket Depth
MBL: Marginal Bone Loss
BMP: Bone Morphogenetic Protein
VEGF: Vascular Endothelial Growth Factor
IGF: Insulin-like growth
PDGF: Platelet-derived growth factor
TGF: Transforming growth factor

## References

[1] Elboraey MO, Alqutaibi AY, Aboalrejal AN, Borzangy S, Zafar MS, Al-Gabri R, Alghauli MA, Ramalingam S. Regenerative approaches in alveolar bone augmentation for dental implant placement: Techniques, biomaterials, and clinical decision-making: A comprehensive review. J Dent. 2025;154:105612. 10.1016/j.jdent.2025.105612. 39909139 10.1016/j.jdent.2025.105612

[2] Abulhassan AH, El Halawani GN, Sweedan OA. Comparison of Autogenous Fresh Demineralized Dentine and Beta-Tricalcium Phosphate in Immediate Implant Placement of Mandibular Molars (Randomized Clinical Controlled Trial). Alexandria Dent J. 2024;49(3):79–86. 10.21608/ADJALEXU.2023.216442.1389.

[3] Dhami B, Shrestha P, Gupta S, Pandey N. Immediate implant placement: current concepts. J Nepal Soc Periodontology Oral Implantology. 2019;3(1):18–24. 10.3126/jnspoi.v3i1.24823.

[4] Bhowal K, Ghosh S. Guided bone regeneration in immediate dental implants - review. Int J Sci Res (IJSR). 2020;9(9):1461–5. 10.21275/SR20919222832.

[5] Kim YK, Ku JK. Guided bone regeneration. J Korean Assoc Oral Maxillofac Surg. 2020;46(5):361–6. 10.5125/jkaoms.2020.46.5.361. 33122463 10.5125/jkaoms.2020.46.5.361 PMC7609932

[6] Goldberg M, Kulkarni AB, Young M, Boskey A. Dentine: structure, composition and mineralization. Front Biosci (Elite Ed). 2011;3(2):711–35. 10.2741/e281. 21196346 10.2741/e281 PMC3360947

[7] Um IW, Ku JK, Lee BK, Yun PY, Lee JK, Nam JH. Postulated release profile of recombinant human bone morphogenetic protein-2 (rhBMP-2) from demineralized dentine matrix. J Korean Assoc Oral Maxillofac Surg. 2019;45(3):123–8. 10.5125/jkaoms.2019.45.3.123. 31334099 10.5125/jkaoms.2019.45.3.123 PMC6620303

[8] Kim KW. Bone Induction by Demineralized Dentine Matrix in Nude Mouse Muscles. Maxillofac Plast Reconstr Surg. 2014;36(2):50–6. 10.4103/jips.jips_62_17. 27489810 10.14402/jkamprs.2014.36.2.50 PMC4281903

[9] Ku JK, Um IW, Jun MK, Kim IH. Dentine-Derived-Barrier Membrane in Guided Bone Regeneration: A Case Report. Mater (Basel). 2021;14(9):2166. 10.3390/ma14092166. 10.3390/ma14092166 PMC8123078 33922832

[10] Choukroun J, Diss A, Simonpieri A, Girard MO, Schoeffler C, Dohan SL, Dohan AJ, Mouhyi J, Dohan DM. Platelet-rich fibrin (PRF): a second-generation platelet concentrate. Part IV: clinical effects on tissue healing. Oral Surg Oral Med Oral Pathol Oral Radiol Endod. 2006;101(3):e56–60. 10.1016/j.tripleo.2005.07.011. 16504852 10.1016/j.tripleo.2005.07.011

[11] Miron RJ, Pikos MA, Estrin NE, Kobayashi-Fujioka M, Espinoza AR, Basma H, Zhang Y. Extended platelet-rich fibrin. Periodontol 2000. 2024;94(1):114–30. 10.1111/prd.12537. 37986559 10.1111/prd.12537

[12] Kawase T, Kamiya M, Kobayashi M, Tanaka T, Okuda K, Wolff LF, Yoshie H. The heat-compression technique for the conversion of platelet-rich fibrin preparation to a barrier membrane with a reduced rate of biodegradation. J Biomed Mater Res B Appl Biomater. 2015;103(4):825–31. 10.1002/jbm.b.33262. 25132655 10.1002/jbm.b.33262

[13] Mizar M, Hassan K, Mohammed A, Mwafy I. Platelet Rich Fibrin Versus Collagen Membrane Combined with Beta Tri Calcium Phosphate/Collagen for Treatment of Dehiscence around Immediately Placed Implants Clinical and Radiographic Comparative Study. Al-Azhar Assiut Dent J. 2021;4(2):189–96. 10.21608/aadj.2021.208221.

[14] Sennerby L, Meredith N. Implant stability measurements using resonance frequency analysis: biological and biomechanical aspects and clinical implications. Periodontol 2000. 2008;47:51–66. 10.1111/j.1600-0757.2008.00267.x. 18412573 10.1111/j.1600-0757.2008.00267.x

[15] Morgulis JR. Indices of periodontal disease. Periodontal Abstr. 1975;23(1):13–20. 146849

[16] Mounir M, Beheiri G, El-Beialy W. Assessment of marginal bone loss using full thickness versus partial thickness flaps for alveolar ridge splitting and immediate implant placement in the anterior maxilla. Int J Oral Maxillofac Surg. 2014;43(11):1373–80. 10.1016/j.ijom.2014.05.021. 24973295 10.1016/j.ijom.2014.05.021

[17] Attia A, Haggag M, Said W, Tawfik M. Evaluation of bone density after bone condensation around immediate loaded dental implants using different techniques. J Amer Sci. 2020;16(7):33–42. 10.7537/marsjas160720.06.

[18] Buser D, Chappuis V, Belser UC, Chen S. Implant placement post extraction in esthetic single tooth sites: when immediate, when early, when late? Periodontol 2000. 2017;73(1):84–102. 10.1111/prd.12170. 10.1111/prd.12170 28000278

[19] Santos PL, Gulinelli JL, Telles Cda S, Betoni Júnior W, Okamoto R, Chiacchio Buchignani V, Queiroz TP. Bone substitutes for peri-implant defects of postextraction implants. Int J Biomater. 2013;2013:307136. 10.1155/2013/307136. 24454377 10.1155/2013/307136 PMC3876702

[20] Gassling V, Purcz N, Braesen JH, Will M, Gierloff M, Behrens E, Açil Y, Wiltfang J. Comparison of two different absorbable membranes for the coverage of lateral osteotomy sites in maxillary sinus augmentation: a preliminary study. J Craniomaxillofac Surg. 2013;41(1):76–82. 10.1016/j.jcms.2012.10.015. 23218506 10.1016/j.jcms.2012.10.015

[21] AlKudmani H, Al Jasser R, Andreana S. Is Bone Graft or Guided Bone Regeneration Needed When Placing Immediate Dental Implants? A Systematic Review. Implant Dent. 2017;26(6):936–44. 10.1097/ID.0000000000000689. 29095788 10.1097/ID.0000000000000689

[22] Cervera-Maillo JM, Morales-Schwarz D, Morales-Melendez H, Mahesh L, Calvo-Guirado JL. Autologous tooth dentine graft: a retrospective study in humans. Med (B Aires). 2021;58(1):56. 10.3390/medicina58010056. 10.3390/medicina58010056 PMC8778028 35056364

[23] Fathy AM, Abd-ElAkher MH, Elfeky AA. Effect of using mineralized dentine particulate grafted for socket preservation. Al-Azhar J Dent Sci. 2019;22(3):211–5. 10.21608/ajdsm.2019.108445.

[24] Dhuvad JM, Mehta D. Does an autogenous demineralized dentine (ADDM) graft has the ability to form a new bone? Natl J Maxillofac Surg. 2021 May-Aug;12(2):181–7. 10.4103/njms.NJMS_12_19. 10.4103/njms.NJMS_12_19 PMC8386253 34483574

[25] Li S, Gao M, Zhou M, Zhu Y. Bone augmentation with autologous tooth shell in the esthetic zone for dental implant restoration: a pilot study. Int J Implant Dent. 2021;7(1):108. 10.1186/s40729-021-00389-w. 34748111 10.1186/s40729-021-00389-w PMC8575770

[26] Awad KAI, Tawik MAM, Hussein MM, El-Farag SAA, Sameaa SESA. Tooth shell versus bone shell technique for horizontal maxillary alveolar ridge augmentation. BMC Oral Health. 2025;25(1):642. 10.1186/s12903-025-05940-4. 40281533 10.1186/s12903-025-05940-4 PMC12032675

[27] Mohamad EHFA, Awad S, Abbas MH, Elsheikh HA. Autogenous dentine-derived membrane as a biologic alternative to collagen membrane for guided bone regeneration in immediate implant placement in the posterior mandible: a randomized clinical trial. Odontology. 2025 Dec;10. 10.1007/s10266-025-01271-6. 10.1007/s10266-025-01271-6 PMC13631035 41372516

[28] Picone A, Castro F, Falcão A, Medina JG, Minetti E, Fernandes JCH, Fernandes GVO. Autogenous Tooth Graft Biomaterial in Guided Bone Regeneration: A Comprehensive Review. Surgeries. 2024;5(4):929–47. 10.3390/surgeries5040075.

[29] Ling Y, Chen D, Li P, Zeng X, Xu W. Repairing alveolar bone defect using demineralized dentine grafts: a meta-analysis of randomized controlled trials. BMC Oral Health. 2024;24(1):1368. 10.1186/s12903-024-05156-y. 39538212 10.1186/s12903-024-05156-y PMC11562794

[30] Mahesh L, Kurtzman GM, Gulati N. Autogenous Tooth-Derived Graft Material in Alveolar Bone Regeneration: A Clinical and Histological Assessment. Med Res Archives. 2025;13(8). 10.18103/mra.v13i8.6785.

[31] Estrin NE, Ahmad P, Tran TB, Espinoza AR, Holmes R, Imber JC, Farshidfar N, Miron RJ. The Evolution of Extended Platelet-Rich Fibrin Membranes for Socket Grafting: Part Two: A Randomized Clinical Trial Comparing These Membranes with Collagen Membranes. Dent J (Basel). 2026;14(1):45. 10.3390/dj14010045. 41590169 10.3390/dj14010045 PMC12840002

[32] Estrin NE, Basma H, Espinoza AR, Pinto MAC, Pikos MA, Miron RJ. Extended Platelet-Rich Fibrin as a Membrane for Lateral Window Sinus Lifts: A Case Series. Clin Implant Dent Relat Res. 2025;27(1):e13427. 10.1111/cid.13427. 39671152 10.1111/cid.13427

[33] Alsabahi H, Mowafey B, Kandil I, Elgohary N, Youssef J. Clinical and radiographic evaluation of autologous albumin-rich platelet-rich fibrin membrane versus synthetic collagen membrane for immediate dental implant placement in the maxillary esthetic zone: a preliminary randomized clinical trial. BMC Oral Health. 2026;26(1):292. 10.1186/s12903-026-07683-2. 41514411 10.1186/s12903-026-07683-2 PMC12903329

[34] Jia K, You J, Zhu Y, Li M, Chen S, Ren S, Chen S, Zhang J, Wang H, Zhou Y. Platelet-rich fibrin as an autologous biomaterial for bone regeneration: mechanisms, applications, optimization. Front Bioeng Biotechnol. 2024;12:1286035. 10.3389/fbioe.2024.1286035. 38689760 10.3389/fbioe.2024.1286035 PMC11058865

[35] Kolerman R, Qahaz N, Barnea E, Mijiritsky E, Chaushu L, Tal H, Nissan J. Allograft and Collagen Membrane Augmentation Procedures Preserve the Bone Level around Implants after Immediate Placement and Restoration. Int J Environ Res Public Health. 2020;17(4):1133. 10.3390/ijerph17041133. 32053928 10.3390/ijerph17041133 PMC7068471

[36] Sayed AJ, Shaikh SS, Shaikh SY, Hussain MA, Tareen SUK, Awinashe V. Influence of Inter-Radicular Septal Bone Quantity in Primary Stability of Immediate Molar Implants with Different Length and Diameter Placed in Mandibular Region. A Cone-Beam Computed Tomography-Based Simulated Implant Study. J Pharm Bioallied Sci. 2021;13(Suppl 1):S484–91. 10.4103/jpbs.JPBS_818_20. 34447139 10.4103/jpbs.JPBS_818_20 PMC8375907

[37] Khalifah SA, Noureldin M, Eldibany M. A comparative study between the effect of platelet rich fibrin and concentrated growth factors on osseointegration of immediate implants (a randomized clinical trial). Alexandria Dent J. 2023;48(2):39–45. 10.21608/adjalexu.2022.136173.1272.

[38] Pirpir C, Yilmaz O, Candirli C, Balaban E. Evaluation of effectiveness of concentrated growth factor on osseointegration. Int J Implant Dent. 2017;3:7. 10.1186/s40729-017-0069-3. 28258471 10.1186/s40729-017-0069-3 PMC5336440

[39] Mohamed AE, El-Mohandes WA, El-Feky AH. Evaluation of effectiveness of concentrated growth factors on osseointegration around immediate dental implant. Al-Azhar Assiut Dent J. 2019;2(2):93–100. 10.21608/aadj.2019.76416.

[40] Monje A, Salvi GE. Diagnostic methods/parameters to monitor peri-implant conditions. Periodontol 2000. 2024;95(1):20–39. 10.1111/prd.12584. 38923148 10.1111/prd.12584

[41] Abdel-Rahman FH, Salem AS, El-Shinnawi UM, Hammouda NI, El-Kenawy MH, Maria OM. Efficacy of autogenous platelet-rich fibrin vs slowly resorbable collagen membrane with immediate implants in the esthetic zone. J Oral Implantol. 2021;47(4):342–51. 10.1563/aaid-joi-D-20-00124. 32870251 10.1563/aaid-joi-D-20-00124

[42] Ali MS, Khalil AA. Evaluation of the effect of using Autogenous Partially Demineralized Dentine Matrix (APDDM) versus xenograft around Immediate Implant (Randomized clinical Study). Minia J Med Res. 2022;33(2):121–6. 10.21608/mjmr.2022.251091.

[43] El Komi HAK, Bayomi MM, Eldestawy MT. Clinical outcome of an injectable platelet-rich fibrin and platelet-rich plasma on immediate dental implant. Al-Azhar J Dent Sci. 2021;24(4):417–24. 10.21608/ajdsm.2020.39073.1096.

[44] Chauhan SR, Kabains R, Sharma A. A comparative evaluation of periodontal parameters around immediate implants with and without platelet-rich fibrin (PRF): a clinical study. IOSR J Dent Med Sci. 2020;19(7):01–13. 10.9790/0853-1907110113.

[45] Saravi BE, Putz M, Patzelt S, Alkalak A, Uelkuemen S, Boeker M. Marginal bone loss around oral implants supporting fixed versus removable prostheses: a systematic review. Int J Implant Dent. 2020;6(1):20. 10.1186/s40729-020-00217-7. 32488421 10.1186/s40729-020-00217-7 PMC7266905

[46] Wu D, Zhou L, Lin J, Chen J, Huang W, Chen Y. Immediate implant placement in anterior teeth with grafting material of autogenous tooth bone vs xenogenic bone. BMC Oral Health. 2019;19:266. 10.1186/s12903-019-0970-7. 31791302 10.1186/s12903-019-0970-7 PMC6889614

[47] Alhaj F, Shokry M, Attia N. The efficiency of using advanced platelet-rich fibrin–autogenous bone graft mixture around immediately placed dental implants in mandibular molar region: a randomized controlled clinical trial. Egypt Dent J. 2023;64:2035–48. 10.21608/edj.2018.76743.

[48] Issa NSH, Othman TA, Sleman BM. A comparative radiographic study of bone density changes around titanium implants in the posterior mandible, preoperative, and postoperative. Ann Med Surg. 2024;86(6):3216–21. 10.1097/MS9.0000000000002142. 10.1097/MS9.0000000000002142 PMC11152818 38846880

